# Impact of Video-Assisted Teaching on Knowledge and Practice Regarding the Use of Metered Dose Inhalers Among Patients With Obstructive Respiratory Disorders

**DOI:** 10.7759/cureus.83374

**Published:** 2025-05-02

**Authors:** Gargee U Karadkar, Rashmi P Choudhary

**Affiliations:** 1 Medical Surgical Nursing, Bharati Vidyapeeth (Deemed to be University) College of Nursing, Navi Mumbai, IND; 2 Medical Surgical Nursing, Masina Hospital Trust, Mumbai, IND

**Keywords:** asthma, copd, knowledge, metered dose inhaler, patient teaching, practice, video-assisted, video-based educational intervention

## Abstract

Introduction:* *Obstructive respiratory disorders include asthma and chronic obstructive pulmonary disease (COPD). This study was conducted with an aim to assess the impact of video-assisted teaching on knowledge and practice regarding the use of metered dose inhalers among outpatient department patients with obstructive respiratory disorders and a comparison of these knowledge and practices across the intervention and control groups was done.

Methodology: A quasi-experimental two-group pre-test and post-test design was used; 162 participants (81 each in the intervention and control groups) were selected using a simple random sampling technique, all from the Navi Mumbai Municipal Corporation Hospital, Navi Mumbai. A pre-test was taken, and video-assisted teaching was provided related to knowledge and practice regarding the use of metered dose inhalers, and on the eighth day, the post-test was conducted. The tools used were a structured questionnaire to assess the knowledge and a checklist to assess practices regarding the use of metered dose inhalers. The analysis was performed using frequency and percentage distribution. The impact of the intervention was statistically evaluated by paired and unpaired t-tests, and the association of pre-interventional knowledge and practice with the demographic variables was tested by the chi-square test.

Result: The results of the present study showed that the pre-test knowledge score for the maximum participants in both the intervention and control groups was poor, i.e., 28 (34.57%) and 26 (32.1%), respectively. The maximum participants scored poorly in the pre-test score for practice regarding the use of metered dose inhalers, i.e., 30 (37.03%) and 36 (44.44%), respectively. There was a significant improvement in the knowledge and practice scores of participants in the intervention group after the intervention of video-assisted teaching. The mean of the pre-test knowledge score was 10.81, the standard deviation (SD) was 8.28, the t-value was -14.65, and the p-value was 0.001. The mean of the pre-test practice score was 11.48, the SD was 4.36, the t-value was -17.97, and the p-value was 0.001. Thus, a statistically significant difference between knowledge and practices regarding the use of metered dose inhalers in the intervention group of patients with obstructive respiratory disorders was found, as calculated by the paired t-test (p-value < 0.05). A comparison of the mean and SD of post-test scores for knowledge and practice, as calculated with the unpaired t-test, showed a statistically significant difference between the intervention and control groups, with p-value < 0.05. There was no significant association between the pre-interventional knowledge score regarding the use of metered dose inhalers among the patients and sociodemographic variables (p > 0.05). Similarly, no significant association was found for practice score with the sociodemographic variables (p > 0.05) except for the variable of the duration of illness.

Conclusion: Findings concluded an improvement in the knowledge and practices of patients with obstructive respiratory disorders regarding the use of metered dose inhalers after the implementation of video-assisted teaching among patients with obstructive respiratory disorders.

## Introduction

Obstructive respiratory disorders include conditions that are characterized by airway obstruction, such as bronchitis, bronchiectasis, asthma, and chronic obstructive pulmonary disease (COPD). Among these, asthma and COPD are the most common diseases that are prevalent globally. In the 21st century, COPD is a disease that has become one of the major health issues in developing countries [1,2]. Common environmental factors like air pollution due to increasing urbanization, long-term smoking, exposure to chemicals, or second-hand smoke predispose patients to suffer from COPD. Asthma is a chronic inflammatory disorder of the lungs in which there is inflammation and narrowing of the airways. The patient may present with seasonal or regular asthma exacerbations [3]. Inhaled medications are used to treat respiratory disorders such as asthma, COPD, and obstructive lung disorders. With the advent of technology and modern medicine, conventional tablets and syrups have been used in conjunction with inhalers for relaxation of the airways to ease breathing. These have been proven to be very effective and safe ways to relieve dyspnea. Inhalers have been found to be patient-friendly, making it easy for them to use and very handy to carry wherever required and available at an affordable cost. Even if the doctors prescribe medicines, the patient needs to follow the instructions for increasing the effectiveness of treatment [4].

Metered dose inhalers (MDIs) are the first-choice device for most people with COPD or asthma with acute exacerbations, but they are not easy to use unless proper education is provided. Studies have shown that an incorrect inhaler technique can lead to inadequate asthma management, resulting in poor asthma control and clinical outcomes. Rates of incorrect inhaler techniques continue to remain high among asthma patients, with minimal improvement in recent times. Since this remains a real challenge in achieving optimal asthma control, investing more time in educating patients on appropriate inhaler use becomes important for healthcare professionals [5].

In practice, patient satisfaction regarding the correct self-use of the MDI device has remained a matter of concern, thus affecting patient outcomes. Nurses are primarily involved in caring for patients and administering the inhaler therapy. Patient education on the correct use of the inhaler technique by nurses would provide confidence to patients on the effective use of inhalers and optimize the relief and control of asthma symptoms. It will further help in reducing complications and subsequent mortality in patients living with asthma or COPD [6].

Thus, this study focused on educating patients by developing and incorporating a video-assisted educational intervention on knowledge and practice regarding the use of MDIs for patients with obstructive respiratory disorders. It aimed to assess the effectiveness of this video-assisted teaching in patients who received the intervention with the help of pre-intervention and post-intervention observations, compare the findings with the control group, and find the association between pre-interventional knowledge and practices with selected sociodemographic variables of the patients.

The hypotheses that were statistically tested were as follows: H01: There is no significant difference between knowledge and practices regarding the use of MDIs in the intervention group of patients with obstructive respiratory disorders before and after video-assisted teaching at a 0.05 level of significance. H02: There is no significant difference between knowledge and practices regarding the use of MDIs between the intervention and control groups of patients with obstructive respiratory disorders at a 0.05 level of significance. H03: There is no significant association between pre-interventional knowledge and practices regarding the use of MDIs among patients with obstructive respiratory disorders and sociodemographic variables at the 0.05 level of significance.

## Materials and methods

Study design

A quantitative research approach and quasi-experimental pre-test post-test design were employed to evaluate the impact of the video-assisted teaching intervention on knowledge and practice regarding the use of MDIs among patients with obstructive respiratory disorders. Participants were assigned to an intervention group and a control group. 

Study participants and procedures

The patients were eligible if they were aged 18 years or older, diagnosed with obstructive respiratory disorders i.e., COPD and asthma and attending chest and pulmonary outpatient department from the selected hospital, received treatment on outpatient basis from Navi Mumbai Municipal Corporation Hospital in Navi Mumbai, using an MDI with or without spacer and who understood Hindi, English, or Marathi language. Patients were excluded if they were on other forms of medication or using MDIs for more than five years or if they did not provide written informed consent.

The data were collected after prior permission from the Ethical Committee of Bharati Vidyapeeth (Deemed to be University) College of Nursing, Pune (BV(DU)/CON/EC/64/ 2021-22) and hospital authorities, followed by informed consent being taken from the patients. A total sample of 162 patients diagnosed with obstructive respiratory disorders was included using a probability sampling method, with a simple random sampling technique for ensuring unbiased representation of the population. The sample size was determined by power analysis to ensure adequate statistical power for the study. The calculated sample size was 162 patients, with 81 participants in the intervention group and 81 participants in the control group. Probability simple random sampling was used for the selection of the participants. The 81 participants who fulfilled the inclusion criteria were randomly assigned to the intervention group and 81 patients to the control group separately. The data were collected on participant demographics, medical information of the participants, and data on knowledge regarding the use of MDIs with a questionnaire comprising 21 multiple-choice questions (see Appendix A). The checklists (Appendix B) were used to observe the practice of the use of MDIs by the participants; two checklists were used for observing the use of MDIs with a spacer (20 steps) and without a spacer (21 steps). The tools for data collection and video were validated by experts. The tools were tested for reliability using kr-20 and Cronbach’s alpha reliability coefficients. The tools were found to be reliable, with a reliability value of 0.75 for the knowledge questionnaire and 0.86 for the checklist.

Intervention 

**Figure 1:**
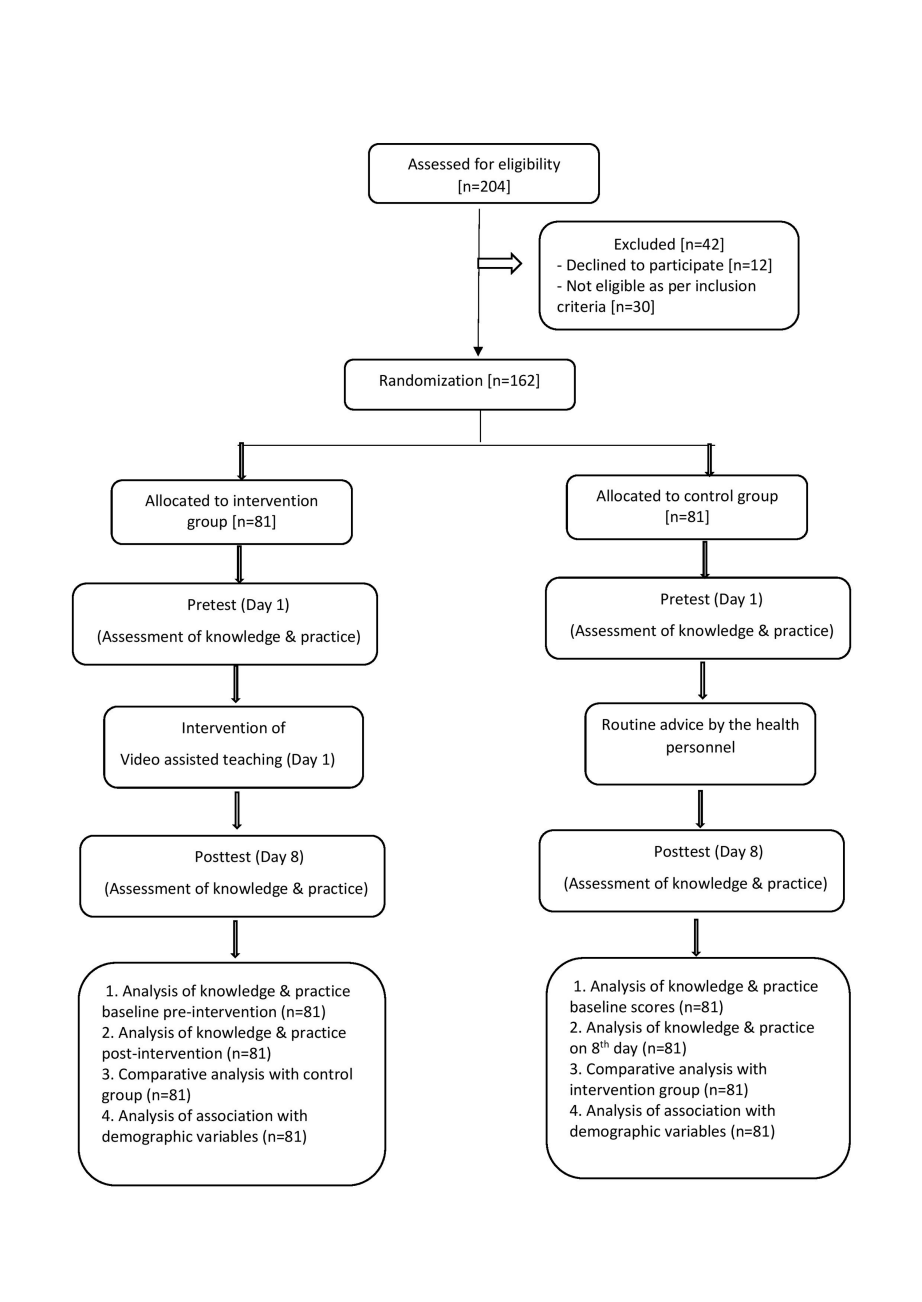
Schematic presentation of the research design

Participants who were eligible as per the inclusion criteria were randomly assigned to the intervention group that received video-assisted teaching intervention on knowledge and practice regarding the use of MDIs and the control group that received routine information from the health personnel, as depicted in Figure 1. The video included a demonstration of an MDI with a spacer and without a spacer with the help of audio-visual aids, such as charts and a PowerPoint presentation. A live commentary of the steps was explained in the video, divided into three parts, i.e., pre-procedure, during procedure, and post-procedure. The videos were made in three languages, viz., English, Hindi, and Marathi. The intervention was provided by a trained nurse researcher; a pre-test was conducted using the knowledge questionnaire and practice checklist. The participants were provided with video-assisted teaching on the same day. A post-test was conducted on the eighth day of the intervention.

Statistical analysis

Data analysis was computed using descriptive and inferential statistics. Participant demographics and medical characteristics were described using frequency and percentage. The comparative statistical analysis was done using a paired t-test, a two-sample t-test, and a chi-square test. In the tests of significance, a p-value less than 0.05 was used. IBM SPSS Statistics for Windows, version 23.0 (released 2015, IBM Corp., Armonk, NY) was used in the analysis.

## Results

A total of 162 participants were included in the intervention group (n = 81) and control group (n = 81). The results are described in sections on frequency and percentage distribution of demographic data, followed by the knowledge and practice levels of the participants in the intervention group and control group. The analysis also includes pre-test and post-test scores assessed before and after the intervention in the intervention group, as well as a comparative analysis depicting the knowledge and practice for both the intervention and control groups.

Table 1 depicts the demographic variables; out of the total participants, the majority were below 30 years of age in both groups. As per gender, the majority were females in the intervention group, i.e., 39 (48.1%), and the control group, i.e., 41 (50.6%). Regarding education, in both groups, the maximum participants had diploma-level education (31 (38.3%) and 32 (39.5%), respectively). The majority in the intervention group were professionals, i.e., 32 (39.5%), and 34 (42%) from the control group were engaged in technical work. The majority belonged to nuclear families from both groups. Thirty-nine (48.1%) participants from the intervention group and 33 (40.7%) participants from the control group had information regarding the use of MDIs. Social media was the most popular source of information as compared to doctors, nurses, or friends.

**Table 1:** Distribution of demographic data according to frequency and percentage N = 81 (intervention group), 81 (control group)

Demographic variable	Category	Intervention group		Control group	
Age		f	%	f	%
	<30 years	42	51.9	40	49.4
	30.1-40 years	23	28.4	23	28.4
	>40.1 years	16	19.7	18	22.2
Gender	Female	39	48.1	41	50.6
	Male	42	51.9	40	49.4
Education	Diploma	31	38.3	32	39.5
	Graduation	8	9.9	8	9.9
	High school	4	4.9	1	1.2
	Intermediate	10	12.3	29	35.8
	Middle school	2	2.5	2	2.5
	Professional degree	26	32.1	9	11.1
Occupation	Clerks	1	1.2	11	13.6
	Craft and related work	0	0	1	1.2
	Professionals	32	39.5	16	19.8
	Technical work	31	38.3	34	42
	Unemployed	17	21	19	23.4
Type of family	Joint	24	29.6	20	24.7
	Nuclear	52	64.2	53	65.4
	Separated family	5	6.2	8	9.9
Information on the use of inhalers	Yes	39	48.1	33	40.7
	No	42	51.9	48	59.3

The medical characteristics of the participants, as shown in Table 2, show that in both groups, 44 (54.3%) participants were diagnosed with asthma and 37 (45.7%) with COPD; the majority were diagnosed with asthma for a duration of more than two to three years. From both groups, the maximum participants did not use MDIs with spacers, and the majority of them were using inhalers for a period of more than one to two years. Regarding the frequency of the use of inhalers, in the intervention group, the majority, i.e., 24 (29.6%), were using inhalers twice a day, whereas 33 (40.8%) in the control group were using inhalers once a day.

**Table 2:** Distribution of medical characteristics according to frequency and percentage N = 81 (intervention group), 81 (control group)

Variable	Category	Intervention group		Control group	
		F	%	F	%
Diagnosis	Asthma	44	54.3	44	54.3
	COPD	37	45.7	37	45.7
Duration of illness	1.1 to 2 years	18	22.2	16	19.8
	2.1 to 3 years	28	34.6	26	32.1
	3.1 to 4 years	15	18.5	24	29.6
	4.1 to 5 years	20	24.6	15	18.5
Use of a metered dose inhaler	With a spacer	10	12.3	2	2.5
	Without a spacer	71	87.7	79	97.5
Duration of use of a metered dose inhaler	1.1 to 2 years	42	51.9	16	19.8
	2.1 to 3 years	13	16.0	18	9.9
	3.1 to 4 years	15	18.5	20	13.6
	4.1 to 5 years	11	13.6	27	6.2
Frequency of use of a metered dose inhaler	Once a day	23	28.4	33	40.8
	Twice a day	24	29.6	26	32.1
	Thrice a day	13	16.1	10	12.3
	More than three times	21	25.9	12	14.8

The pre-test knowledge regarding the use of an MDI among patients with obstructive respiratory disorders before video-assisted teaching, as presented in Table 3, shows that in both the intervention and control groups, the majority of participants had poor knowledge, i.e., 28 (34.57%) and 26 (32.1%), respectively.

**Table 3:** Pre-test knowledge scores regarding use of metered dose inhaler among participants N = 81 (intervention group), 81 (control group)

Knowledge level	Score category	Group			
		Intervention		Control	
		Frequency	Percentage	Frequency	Percentage
Poor	1-6 (0-25%)	28	34.57	26	32.1
Average	7-12 (26-50%)	20	24.69	18	22.22
Good	13-18 (51-75%)	15	18.52	20	24.70
Very good	19-24 (76-100%)	18	22.22	17	20.98
Total		81	100	81	100

The pre-test practice regarding the use of MDIs among patients with obstructive respiratory disorders before video-assisted teaching, as depicted in Table 4, in both the intervention and control groups, show that majority had poor practice scores, i.e., 30 (37.03%) and 36 (44.44%), respectively. Participants from both groups had scored low for the category of very good practices, i.e., 13 (16.05%) and 12 (14.81%) in the intervention and control groups, respectively.

**Table 4:** Pre-test practice scores regarding the use of metered dose inhalers among the participants N = 81 (intervention group), 81 (control group)

Practice level	Score category	Group			
		Intervention		Control	
		Frequency	Percentage	Frequency	Percentage
Poor	0-4 (0-25%)	30	37.03	36	44.44
Average	5-10 (26-50%)	18	22.22	16	19.75
Good	11-15 (51-75%)	20	24.70	17	21
Very good	16-21 (76-100%)	13	16.05	12	14.81
Total		81	100	81	100

The knowledge scores of the pre-test and post-test after the intervention as presented in Figure 1 shows that in the intervention group, in the pre-test, 28 (34.57%) participants had poor knowledge, 20 (24.69%) participants had average knowledge, 15 (18.52%) participants had good knowledge, and 18 (22.22%) had very good knowledge. In the post-test, 17 (20.90%) participants had poor knowledge, 16 (19.90%) participants had average knowledge, 26 (32.20%) participants had good knowledge, and 22 (27%) participants had very good knowledge. The majority of participants had good knowledge after the intervention.

**Figure 2:**
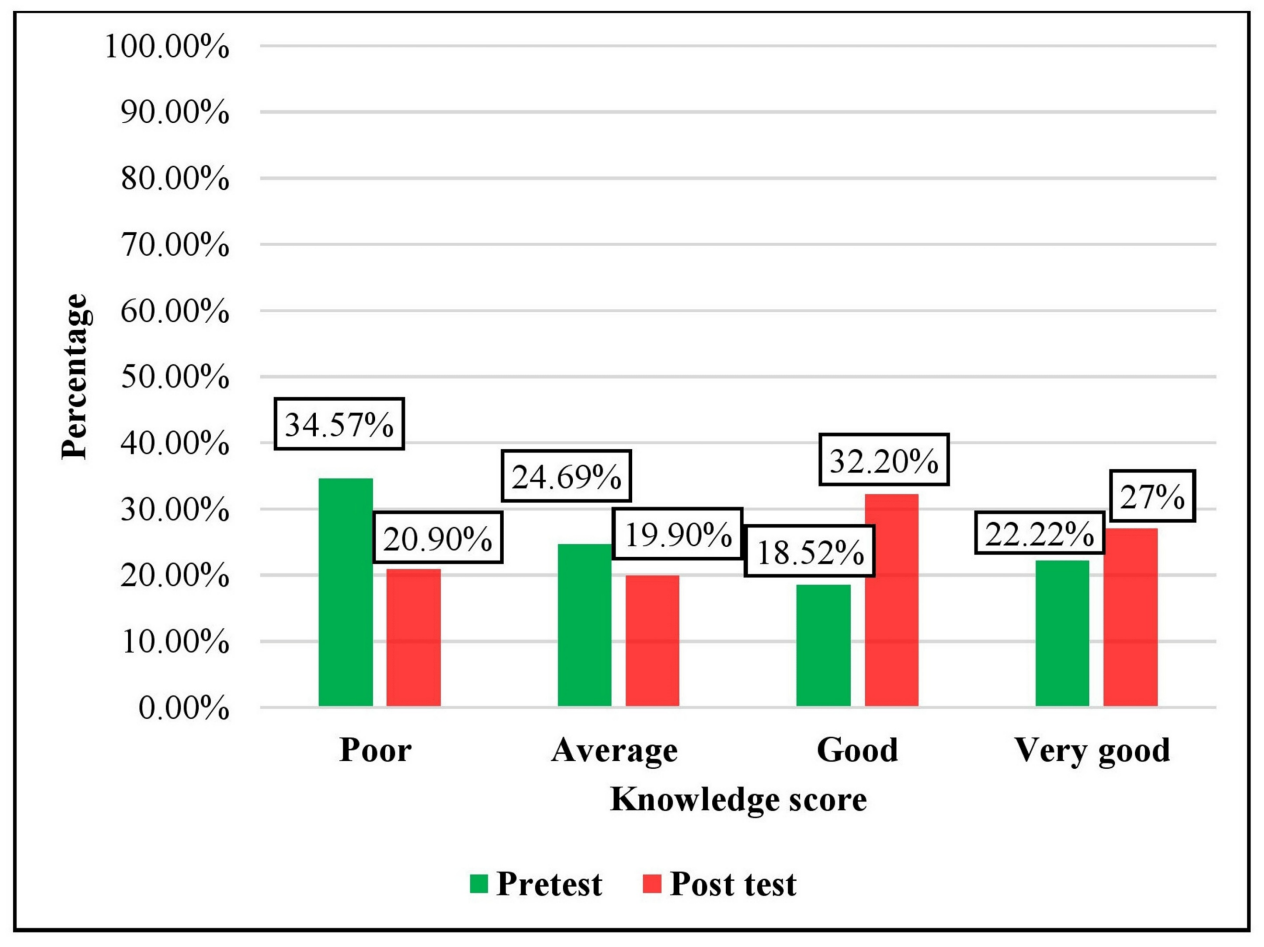
Distribution of pre-test and post-test knowledge scores before and after video-assisted teaching in the intervention group N = 81 (intervention group), 81 (control group)

The practice scores of the pre-test and post-test before and after the intervention are presented in Figure 2. In the intervention group, pre-test scores showed that 30 (37.03%) participants had poor scores, 18 (22.22%) participants had average scores, 20 (24.7%) participants had good scores, and 13 (16.05%) participants had very good scores. In the post-test, maximum, i.e., 28 (34.7%), scored good, 26 (32%) scored very good, whereas 15 (18.5%) scored average and 12 (14.8%) scored poor on the scale for practices.

**Figure 3:**
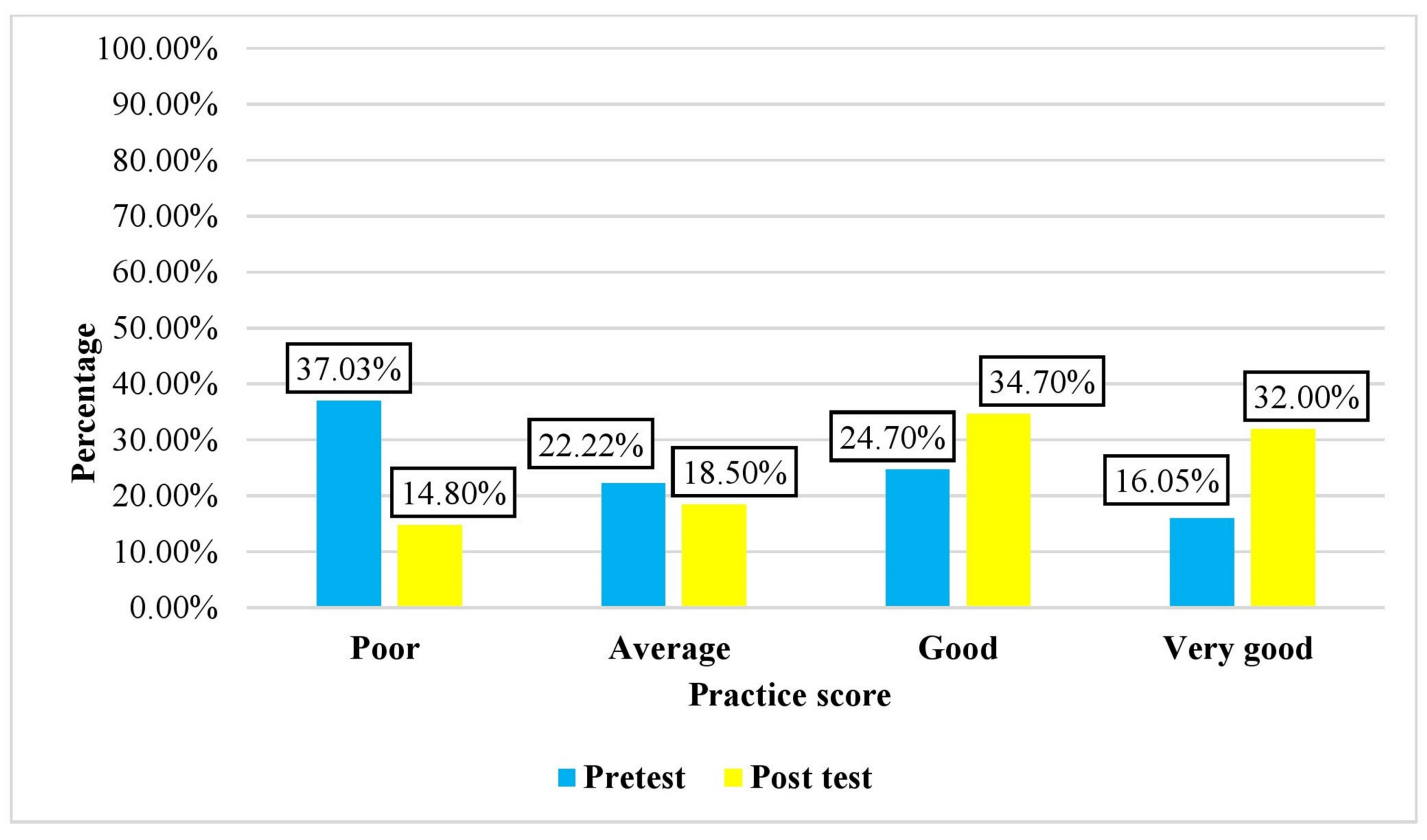
Distribution of the pre-test and post-test practice scores before and after video-assisted teaching in the intervention group N = 81 (intervention group), 81 (control group)

The effectiveness of video-assisted teaching on knowledge and practices regarding the use of MDIs in the intervention group of patients with obstructive respiratory disorders is depicted in Table 5. The mean of the pre-test knowledge score was 10.81, the SD was 8.28, the t-value was -14.65, and the p-value was 0.001. The mean of the pre-test practice score was 11.48, the SD was 4.36, the t-value was -17.97, and the p-value was 0.001. Thus, there was a significant difference between knowledge and practices regarding the use of MDIs in the intervention group of patients with obstructive respiratory disorders before and after the intervention at the 0.05 level of significance as calculated by the paired t-test. The p-values for both knowledge and practice were less than 0.05. Hence, the null hypothesis that no significant difference between the pretest and post-test scores for knowledge and practice regarding the use of MDIs exists was rejected.

**Table 5:** Effectiveness of video-assisted teaching on knowledge and practices regarding the use of metered dose inhalers in the intervention group of patients with obstructive respiratory disorders using the paired t-test at the 0.05 level of significance. N = 81 (intervention group), 81 (control group)

Intervention group	N	Statistic	Pre-test score	Post-test score	Df	t -value	p -value	Significance
Knowledge	81	Mean	10.81	13.88	80	-14.65	0.001	Significant
		Standard deviation	8.28	4.36				
Practice	81	Mean	11.48	15.67	80	-17.97	0.001	Significant
		Standard deviation	8.86	4.71				

Table 6 depicts a comparison between the knowledge of the intervention and control groups that was calculated with an unpaired t-test. The post-test mean of the intervention group was 13.88, and the SD was 4.36; the post-test mean of the control group was 14.72 and the SD was 8.72. The t-value was -19.43 and the p-value was 0.001. The null hypothesis that there was no significant difference between knowledge regarding the use of MDIs among the intervention and control groups of patients with obstructive respiratory disorders at the 0.05 level of significance was rejected, as the p-value < 0.05. 

**Table 6:** Comparison of the knowledge regarding the use of metered dose inhalers among the intervention and control groups of patients with obstructive respiratory disorders using an unpaired t-test at the 0.05 level of significance N = 81 (intervention group), 81 (control group)

Group	N	Mean	Standard deviation (SD)	t-value	df	p-value	Significance
Intervention	81	13.88	4.36	-19.43	80	0.001	Significant
Control	81	14.72	8.72				

Table 7 represents a comparison of practices regarding the use of inhalers between the intervention and control groups was calculated with an unpaired t-test. The post-test mean of the intervention group was 15.67 and the SD was 4.36, and the post-test mean of the control group was 7.91 and the SD was 5.6. The t-value was -18.7 and p-value was 0.001, which was significant at the 0.05 level of significance. Thus, there was a statistically significant difference in both groups (p < 0.05). The null hypothesis that there was no significant difference between practice regarding the use of MDIs among the intervention and control groups of patients with obstructive respiratory disorders at the 0.05 level of significance was rejected. 

**Table 7:** Comparison of the practices regarding the use of metered dose inhalers among the intervention and control groups of patients with obstructive respiratory disorders using an unpaired t-test at the 0.05 level of significance N = 81 (intervention group), 81 (control group)

Group	N	Mean	Standard deviation (SD)	t-value	df	p-value	Significance
Intervention	81	15.67	4.36	-18.7	80	0.001	Significant
Control	81	7.91	5.6				

There was a significant difference between the intervention and control groups of patients with obstructive respiratory disorders for knowledge and practices regarding the use of MDIs at the 0.05 level of significance; thus, the null hypothesis was not accepted.

The association between pre-interventional knowledge and practices regarding the use of MDIs among patients with obstructive respiratory disorders with selected sociodemographic variables was calculated using a chi-square test. The association between pre-interventional knowledge of patients and their demographic variables of age (years), gender, occupation, education, type of family, information regarding the use of MDIs earlier, source of information, diagnosis, duration of illness, use your inhaler, its duration of use, and frequency of use; no significant association was found as p > 0.05. Similarly, no significant association was found between the pre-interventional practices of patients and their demographic variables (p > 0.05) except for the variable of duration of illness. Thus, the null hypothesis was accepted for knowledge and for practices except for the variable of duration of illness (p = 0.036).

## Discussion

Utilization of videos for teaching improves comprehension, cognitive ability, and skills [7]. When used in patient education, they are effective tools for their understanding about the health conditions, and compliance to the treatment is improved as it includes visual and audio explanations. This study incorporated a video to educate patients with obstructive respiratory disorders about MDIs. The intervention group demonstrated a significant improvement in knowledge and practice about its use, as evidenced by improved knowledge and practice scores post-intervention. A comparison of the mean scores of both the intervention and control groups showed a significant difference between the two groups, with the unpaired t-test values and p < 0.05 rejecting the null hypotheses. The findings were relatable to studies that have focused on similar strategies to have improved patient outcomes.

To overcome the problem of incorrect use of inhalers, Al-Kharouf et al. (2023) did a prospective and randomized controlled trial involving 103 asthmatic patients: 51 in the intervention group and 52 in the control group. The patients received intervention of a video-based teach-to-goal strategy, whereas the control group received a verbal education. There was an improvement in the use of inhaler technique in the intervention group (93.4%) in comparison to the control group (49.5%) (p < 0.05). The quality of life was significantly improved at a follow-up assessment [8].

A comparative study by S. F. Tafti et al. (2019) on individual and traditional bedside MDI use and group teaching with video demonstration involved 32 patients who received traditional teaching and 60 patients who received video teaching. In the traditional group, the mean age was 53 years and 20 were males. In the group that received demonstration of videos, the mean age was 62 years and 54 were males. The two groups did not significantly differ in the correct explanation of MDI use by phone interviews (p > 0.05). However, the two groups did statistically differ in age group and Saint George Respiratory Questionnaire scores at the beginning of the study (p < 0.004) [9].

Interestingly, video-based education has proved effective in patients with other health conditions too. A study by E. R. Miller et al. (2021) conducted a quality improvement program at a primary care clinic in Saudi Arabia that enrolled 269 adults with elevated levels of blood pressure who were not prescribed anti-hypertensive medications and utilized short, animated videos, as well as web-linked diet lifestyle intervention. Of the participants, 77% were males and 33% were females, and the average age was 41.6 years. A high level of engagement was demonstrated after the intervention. Those who return demonstrated a considerable reduction in systolic BP levels, i.e., -10.5 mm Hg from the baseline readings of 138.0 (7.2) mm Hg, which was a significant change (p < 0.001) [10].

In an article by N. Balasubramanian et al. (2018) on evaluating the implementation of a VATM, i.e., video-assisted teaching module regarding home care of schizophrenic patients, the authors provided intricate steps and process details of the preparation of the teaching material. Such a module demonstrated a reduction in burden of the caregivers with improved patient care, effective coping, and adjustment with developing good skills in patient care at home [11].

The review of the literature mentions the use of novel techniques other than the written or verbal methods of patient education improves patient adherence to the therapies and has better health outcomes [12]. Mastering the appropriate inhaler technique is directly correlated to better control of the clinical symptoms and reduced exacerbations of the disease. The results of the study go in line with the findings of the studies done on patients with obstructive respiratory disorders and on those with other chronic conditions like hypertension or psychiatric conditions. The explanation about the use of the inhaler in patient-friendly language made it more comprehensible for the patients understanding. The one-to-one interaction with the patients also helped in better engagement of the patients, which was evident from the practice score that was assessed with a checklist that checked for the steps followed sequentially as per the appropriate and effective use of the inhaler device and had a better learning outcome. Future opportunities could be researching therapy adherence aspects by utilizing qualitative methodologies and conducting randomized clinical trials with the use of modern patient teaching technologies. 

The researchers do identify certain limitations of the study. The study included only one follow-up observation of the demonstration of MDIs in patients with obstructive respiratory disorders. Thus, repeated teaching, self-motivation levels of patients, and patient self-care education were not included. The patients visiting outpatient department were only included in the study and were from the geographical location of Navi Mumbai. Thus, the findings may not be generalized.

## Conclusions

The results of the study showed a notable improvement in both knowledge and practice regarding the use of MDIs in the patients from the intervention group. Thus, the intervention was found to be a useful aid for better understanding the technique of using MDIs. The findings highlight a positive impact of the video-assisted teaching regarding the knowledge and practice of MDIs, as found by the statistically significant differences between the pre-test and post-test scores among the intervention group. Thus, it can be concluded that patient teaching utilizing both audio and visuals has a greater impact as compared to routine health education. However, no significant association was found between the pre-interventional knowledge of patients and their demographic variables of age (years), gender, occupation, education, type of family, information regarding the use of MDIs earlier, source of information, diagnosis, duration of illness, use of inhaler, its duration of use, and frequency of use. No significant association was found between the pre-interventional practices of patients and their demographic variables except for the variable of duration of illness. Thus, the null hypothesis was accepted for knowledge and for practices except for the variable of duration of illness.

Patient education can be enriched for chronic pulmonary patients and asthmatics, particularly in using medications via inhalers for achieving control of the symptoms and better pulmonary compliance. This study examined the impact of video-assisted teaching of the correct technique of the use of an MDI that was developed by a nurse who plays a pivotal role in patient care and educating them for better adherence to the treatment. The findings suggest potential for the cost-effective use of newer technologies, especially when there is easy availability of mobile-based technology to all globally. The study highlights the need for nurses to use newer and more interactive methods to improve the benevolence, adherence to treatment, and better quality of life of patients having chronic obstructive respiratory disorders.

## Appendices

Questionnaire to assess knowledge regarding the use of metered dose inhalers

Code No:  

Instructions: 

1)      Read the following question carefully and answer it appropriately

2)      Tick (√) the correct answer

3)      All the questions must be answered

4)      Use blue/ black ball point pen only

5)      The information provided will be kept confidential and it will not be used for any  other purpose

Section I  Demographic Data:

**Table 8:** Tool for demographic Variables

1	Age In (Years): ------------		
2	Education:		
	2.1	Primary	
	2.2	Middle School	
	2.3	High School	
	2.4	Intermediate/Diploma	
	2.5	Graduate	
	2.6	Professional Degree	
3	Occupation:		
	3.1	Professionals	
	3.2	Technicians And Associate Professionals	
	3.3	Clerks	
	3.4	Skilled Workers And Shop & Market Sales Workers	
	3.5	Skilled Agricultural & Fishery Workers	
	3.6	Craft And Related Trade Workers	
	3.7	Plant & Machine Operators And Assemblers	
	3.8	Elementary Occupation	
	3.9	Unemployed	
4	Type Of Family:		
	4.1	Nuclear	
	4.2	Joint	
	4.3	Extended	
	4.4	Extended Nuclear	
	4.5	Separated Family	
5	Have You Received Information Regarding Use Metered Dose Inhaler?		
	5.1	Yes	
	5.2	No	
6	If Yes, Then What Is The Source Of Information:		
	6.1	Doctor	
	6.2	Nurse	
	6.3	Any Family Member	
	6.4	Friends	
	6.5	Television	
	6.6	Social Media	

Section II  Medical Information of the Patient

**Table 9:** Medical information of the patient

1	Diagnosis:		
	1.1	COPD	
	1.2	Asthma	
	1.3	Any Other Co-Morbidity Mention	
2	Duration Of Illness		
	2.1	Up To 1 Year	
	2.2	1.1 To 2 Years	
	2.3	2.1 To 3 Years	
	2.4	3.1 To 4 Years	
	2.5	4.1 To 5 Years	
3	How Do You Use Your Inhaler?		
	3.1	Metered Dose Inhaler Without Spacer	
	3.2	Metered Dose Inhaler With Spacer	
4	Use Of Inhaler Since		
	4.1	Less Than 1 Year	
	4.2	1.1 – 2 Years	
	4.3	2.1 – 3 Years	
	4.5	More Than 3 Years	
5	Frequency Of Use Of Inhaler		
	5.1	Once In A Day	
	5.2	Twice In A Day	
	5.3	Thrice In Day	
	5.4	More Than Three Times	

*Section III  Knowledge on the Use of Metered Dose Inhaler*s

**Table 10:** Questionnaire on knowledge regarding use of Metered Dose Inhaler

1	What Is Normal Level Of Oxygen In Body?		
	1.1	80-85%	
	1.2	58-60%	
	1.3	95-100%	
	1.4	75-85%	
2	The Device Used To Relieve The Difficulty In Breathing By Inhaling The Drug Is Called As		
	2.1	Metered Dose Inhaler	
	2.2	Dry Powder Inhaler	
	2.3	Steamer	
	2.4	Nebulizer	
3	The Inhaled Drugs Have A Direct Action On :		
	3.1	Airways	
	3.2	Lungs	
	3.3	Liver	
	3.4	Brain	
4	What Is One Of The Check Points Before Using MDI?		
	4.1	Expiry Date	
	4.2	Pressure	
	4.3	Volume	
	4.4	Colour Of Puff	
	4.5	Name Of Drug	
	4.6	Dosage Of Drug	
5	How To Check Whether The MDI Is Full Or Empty Of Medication?		
	5.1	By Checking Canister	
	5.2	By Checking Use Of Puff	
	5.3	By Checking Weight Of Inhaler	
	5.4	By Shaking The Inhaler	
6	Why Do You Shake Inhaler Before Use?		
	6.1	Mixes Medication Evenly	
	6.2	To Check Whether Medicine Is Filled In Inhaler	
	6.3	To Hear The Sound Of Medicine	
	6.4	To Check Inhaler Is In Working State	
7	Does One Need To Spray One Or More Puffs Into The Air Every Time Before Use ?		
	7.1	Yes	
	7.2	No	
	7.3	Never	
	7.4	Sometimes	
8	Spraying One Or More Puffs Into The Air Before Helps To Assure		
	8.1	Inhaler Is Not Working	
	8.2	Inhaler Is Ready To Use	
	8.3	Measures The Medication	
	8.4	To Check Puff	
9	Tick (√) The Correct Answer Which Part Of Inhaler Does Spacer Is Attached?		
	9.1	Mouth Piece	
	9.2	Mouth Cap	
	9.3	Canister	
	9.4	Counter Dose	
10	The Placement Of Mouth Piece Of The Inhaler Should Be-----------		
	10.1	Between Teeth And Lips (Sealed Tightly)	
	10.2	Between Tongue Lose Around It	
	10.3	Below Tongue	
	10.4	Above Tongue	
11	When Do You Press Down On The Top Of The Metal Canister Once, To Release The Medicine Into The Spacer?		
	11.1	After 1 min	
	11.2	After 2 min	
	11.3	After 3 min	
	11.4	After 4 min	
12	For How Long You Should Hold Your Breath After Inhalation Of Drug?		
	12.1	10 Sec	
	12.2	8 Sec	
	12.3	12 Sec	
	12.4	14 Sec	
13	Holding Of Breath After Inhaling The Drug Is Necessary For Following Reasons?		
	13.1	It Is Waiting Period Drug To Act	
	13.2	Allow Drug To Reach Airways	
	13.3	Allow Drug To Reach In Mouth	
	13.4	Allow Drug To Reach In Lungs	
14	The Reason Of Rinsing Mouth After Taking Corticosteroids Via Metered Dose Inhaler?		
	14.1	To Remove Residual Medication From The Mouth And Throat	
	14.2	To Prevent Nosocomial Infection	
	14.3	To Avoid Taste Of Medicine	
	14.4	To Avoid Smell Of Medicine	
15	What Action Needs To Be Taken After The Complete Use Of Medication?		
	15.1	Reuse Inhaler	
	15.2	Discard The Inhaler	
	15.3	Refill Inhaler	
	15.4	Recycle Inhaler	
16	Is It Needed To A Keep Count Of Puff That You Have Would Be Using?		
	16.1	Always Required	
	16.2	May Be Required	
	16.3	Never Required	
	16.4	Sometimes Required	
17	Which Is The Best Place To Keep The Inhaler?		
	17.1	Fridge	
	17.2	Always Beside You	
	17.3	Drawer	
	17.4	In Your Bag	
18	How Frequently Inhaler Should Be Cleaned?		
	18.1	Every Day	
	18.2	Every Week	
	18.3	Every Time Before Using And After Use	
	18.4	Never	
19	Inhaler Should Be Cleaned With Following		
	19.1	Rinse Under Water	
	19.2	Wash With Soap	
	19.3	Clean With Wet Tissue	
	19.4	Wash With Detergent	
20	Colour Of Counter Dose Inhaler Change From Green To Red What Does It Indicate?		
	20.1	Minimum Puff Are Remaining	
	20.2	Maximum Puff Are Remaining	
	20.3	No Puffs Left	
	20.4	Inhaler Is Not Working	
21	What Are The Following Is Not The Advantages Of Using MDI?		
	21.1	Easy To Use	
	21.2	Not Able To Use	
	21.3	No Drug Preparation Is Needed	
	21.4	Quick To Use Than Other Device.	

Checklist to assess the practice of using metered dose inhaler without spacer

(The investigator will observe the demonstration of use of metered dose inhaler by the patient and mark the inappropriate box)

Participant code:             

Pre-test demonstration:   

Post-test demonstration: 

**Table 11:** Checklist to assess the practice of using metered dose inhalers without a spacer The grading of practices will be done as follows: Yes – 1  No – 0.

Step no	Steps of use of metered dose inhaler	Yes	No
1	Checks the name of the drug and expiry date		
2	Removes the cap from the MDI and chamber		
3	Checks for the cleanliness of the mouthpiece		
4	Shakes the inhaler well for 10 to 15 times		
5	Places the mouthpiece of the chamber between his/her teeth		
6	Holds the MDI canister in an upright position		
7	Seals his/her lips tightly around it		
8	Ensures the mask has a good seal against his/her cheeks and chin.		
9	Ensures that there is no space between the mask and the skin		
10	Takes a breath and the breathes out completely		
11	Presses the canister once		
12	Breathes in slowly and completely through his/her mouth.		
13	Checks if he/she hears a "horn-like" sound that indicates fast breathing so slows down		
14	Holds breath for 10 seconds (counts to 10 slowly) to allow the medication to reach the airways of the lung		
15	Repeats the above steps for each puff ordered by the doctor		
16	Waits about 1 minute in between puffs		
17	Replaces the cap on the MDI when finished		
18	If you are inhaling a steroid, rinses his/her mouth out with water, swish, gargle and spit.		
19	Clean the inhaler after its use		

Checklist to assess the practice of using metered dose inhalers with a spacer

(The investigator will observe the demonstration of use of metered dose inhaler by the patient and mark the inappropriate box)

Participant code:              

Pre-test demonstration   

Post-test demonstration

**Table 12:** Checklist to assess the practice of using metered dose inhalers with a spacer The grading of practices will be done as follows: Yes – 1  No – 0.

Step no	Steps of use of metered dose inhaler	Yes	No
1	Checks the name of the drug and expiry date		
2	Removes the cap from the MDI and chamber		
3	Checks for the cleanliness of the mouthpiece		
4	Shakes the inhaler well for 10 to 15 times		
5	Inserts the MDI into the open end of the chamber of spacer (opposite the mouthpiece).		
6	Places the mouthpiece of the chamber between his/her teeth		
7	Holds the MDI canister in an upright position		
8	Seals his/her lips tightly around it		
9	Ensures the mask has a good seal against his/her cheeks and chin.		
10	Ensures that there is no space between the mask and the skin		
11	Takes a breath and the breathes out completely		
12	Presses the canister once		
13	Breathes in slowly and completely through his/her mouth.		
14	Checks if he/she hears a "horn-like" sound that indicates fast breathing so slows down		
15	Holds breath for 10 seconds (counts to 10 slowly) to allow the medication to reach the airways of the lung		
16	Repeats the above steps for each puff ordered by the doctor		
17	Waits about 1 minute in between puffs		
18	Replaces the cap on the MDI when finished		
19	If you are inhaling a steroid, rinses his/her mouth out with water, swish, gargle and spit.		
20	Clean the inhaler after its use		
